# Cellular origins and evolutionary dynamics of plasma cfDNA in solid tumors: integrating germline, tumor-derived, and hematopoietic variation for MRD and treatment guidance

**DOI:** 10.3389/fcell.2026.1928945

**Published:** 2026-10-14

**Authors:** Quanneng Zhao, Chunlei Wu, Xiao Huang, Miyuan Yang, Bing Zhang

**Affiliations:** 1 Department of Clinical Laboratory, Beijing Anzhen Nanchong Hospital of Capital Medical University and Nanchong Central Hospital, Nanchong, Sichuan, China; 2 Department of Blood Transfusion, Beijing Anzhen Nanchong Hospital of Capital Medical University and Nanchong Central Hospital, Nanchong, Sichuan, China; 3 Department of Clinical Laboratory, Nanchong Hospital of Traditional Chinese Medicine, Nanchong, Sichuan, China

**Keywords:** cell-free DNA, circulating tumor DNA, clonal hematopoiesis, germline variant, molecular residual disease, precision oncology, somatic mutation, variant-source attribution

## Abstract

Plasma cell-free DNA (cfDNA) is a heterogeneous and rapidly changing biological pool derived from malignant cells, normal tissues, and hematopoietic cells. The abundance and molecular composition of circulating tumor DNA (ctDNA) are determined not only by tumor burden but also by tissue architecture, vascular access, cell-death programs, chromatin organization, nuclease processing, systemic clearance, treatment exposure, and clonal evolution. These biological determinants shape the mutation, methylation, fragmentation, nucleosomal, and temporal features exploited by contemporary liquid-biopsy assays. Accurate clinical interpretation therefore requires both sensitive detection and reliable source attribution, particularly the distinction of tumor-derived variants from constitutional germline alterations and clonal hematopoiesis. In this review, we integrate the biological origins and processing of plasma cfDNA with analytical strategies for low-tumor-fraction detection and critically evaluate the evidence supporting postoperative molecular residual disease (MRD), recurrence surveillance, treatment-response assessment, resistance monitoring, and ctDNA-guided therapy across solid tumors. Particular emphasis is placed on distinguishing prognostic validity, treatment-predictive value, and demonstrated clinical utility. Current evidence is strongest in colorectal cancer and, more recently, ctDNA-selected urothelial cancer, whereas routine ctDNA-directed intervention in resected non-small cell lung cancer and breast cancer remains insufficiently validated. By linking cfDNA biology to an explicit hierarchy of clinical evidence, this review defines both the established applications and the major limitations that must be addressed before broader implementation of ctDNA-guided precision oncology.

## 1 ctDNA as a dynamic biomarker in solid tumors

Clinical management of solid tumors continues to rely on anatomical stage, pathological risk factors, imaging, and selected circulating protein biomarkers. These approaches remain indispensable but provide intermittent measurements of disease and may not capture molecular changes that occur before macroscopic progression becomes detectable. Plasma ctDNA offers a complementary, repeatedly accessible biomarker capable of reflecting residual disease, treatment response, clonal selection, and emerging resistance through tumor-associated mutations, copy-number alterations, methylation profiles, and fragmentation features (Gouda et al., 2024; Diehl et al., 2008; Wan et al., 2017).

However, ctDNA should not be regarded as a direct quantitative surrogate for viable tumor mass. Plasma cfDNA represents a composite analyte whose concentration and molecular composition reflect the balance between DNA release, nuclease processing, tissue access to the circulation, and systemic clearance. Tumor histology, disease site, treatment-related cell death, inflammatory injury, and the contribution of nonmalignant hematopoietic cells can therefore substantially modify the measured signal independently of changes in tumor volume (Diehl et al., 2008; Wan et al., 2017). This biological complexity becomes particularly important in MRD settings, where the tumor-derived fraction may approach the analytical detection limit.

Three levels of evidence are consequently distinguished throughout this review. Prognostic validity refers to an association between ctDNA status and subsequent clinical outcome. Treatment-predictive value indicates that ctDNA identifies patients with differential benefit from a particular therapy. Clinical utility requires prospective evidence that treatment decisions based on ctDNA improve clinically meaningful outcomes or safely reduce treatment exposure (Widman et al., 2024). Although postoperative ctDNA has consistently demonstrated strong prognostic validity across several solid tumors, randomized evidence supporting ctDNA-directed management remains restricted to specific clinical settings (Pascual et al., 2022; Verschoor et al., 2024; Kobayashi et al., 2025).

The present review therefore evaluates cfDNA biology, source attribution, analytical performance, and clinical evidence within a single framework, with particular attention to where observational association ends and actionable evidence begins. The clinical continuum of ctDNA applications and their current evidence boundaries are summarized in Figure 1.

**FIGURE 1:**
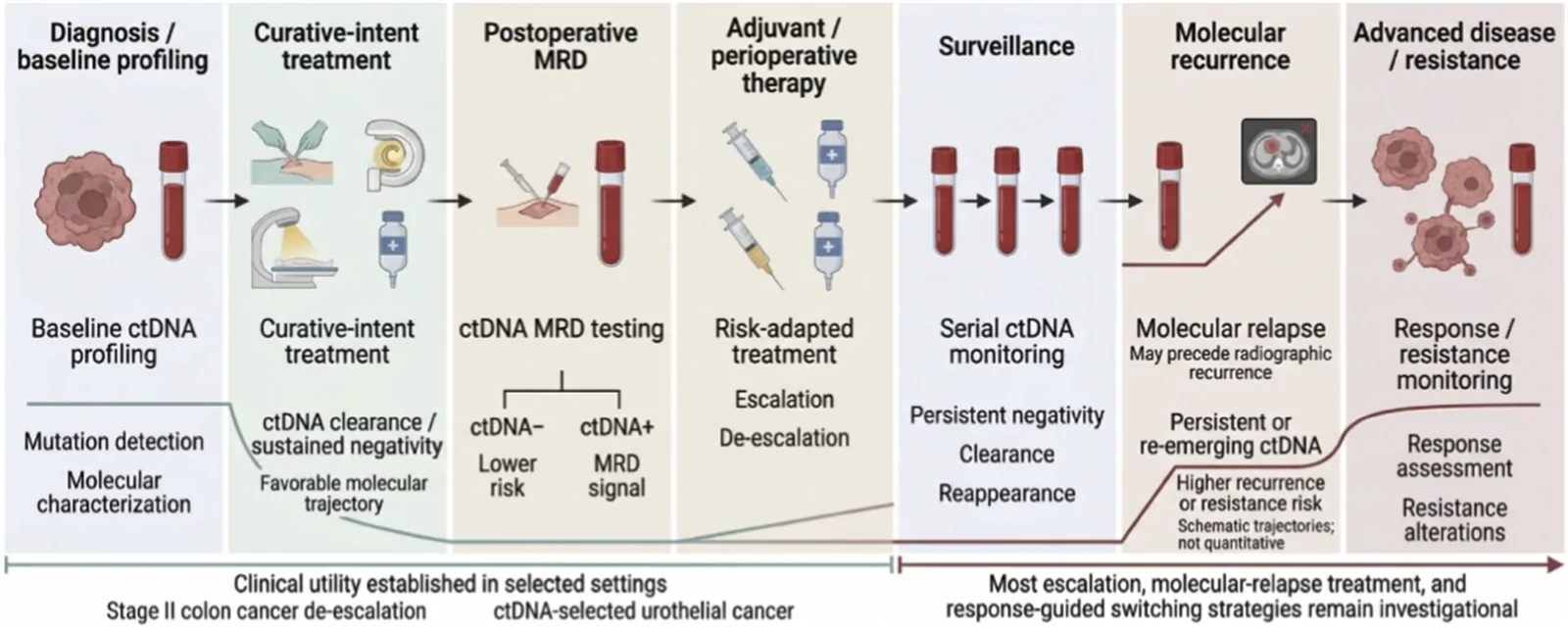
ctDNA across the solid-tumor clinical continuum and its current evidence boundaries. ctDNA can provide complementary molecular information after curative-intent treatment, during adjuvant therapy, surveillance, molecular recurrence, and advanced disease. Postoperative positivity identifies residual-disease risk, whereas longitudinal clearance, persistence, or reappearance provides dynamic information on treatment response and relapse. Importantly, ctDNA-guided treatment has demonstrated clinical utility only in selected settings, and molecular positivity or negativity should be interpreted together with tumor type, assay performance, imaging, pathology, and clinical context. Created in BioRender. Zhang, B. (2026) https://BioRender.com/v4c8cqu.

### 1.1 Review scope and evidence framework

This review prioritizes original biological studies defining cfDNA generation and source attribution, prospective longitudinal MRD cohorts, randomized or prospectively ctDNA-selected interventional trials, analytical-validation studies, and major professional recommendations. Particular emphasis is placed on studies reporting quantitative clinical outcomes, including sample size, recurrence risk, hazard ratios, molecular lead time, ctDNA clearance, and treatment effects. Evidence from retrospective biomarker analyses, prospective observational studies, and randomized intervention trials is considered separately to avoid equating biomarker association with demonstrated clinical utility. Representative studies, sampling strategies, and evidence boundaries for ctDNA applications across solid tumors are summarized in Table 1.

**TABLE 1:** Evidence level, representative sampling strategy, and interpretation boundaries for ctDNA applications in solid tumors.

Study/tumor	Design and cohort	Clinical setting	Representative quantitative result	Evidence supported	Ref.
Reinert et al., CRC	Prospective cohort, n = 125	Postoperative MRD and surveillance	Day-30 ctDNA+: HR 7.2 for recurrence; longitudinal ctDNA+: HR 43.5; mean lead time 8.7 months	Prognostic validity/surveillance	Reinert et al. (2019)
GALAXY, CRC	Prospective observational, n = 2,240	Postoperative MRD	MRD positivity: DFS HR 11.99; OS HR 9.68; sustained vs. transient clearance: 24-month DFS 89.0% vs. 3.3%	Strong prognostic validity	Nakamura et al. (2024)
DYNAMIC, stage II colon	Randomized, n = 455	Adjuvant de-escalation	Chemotherapy 15% vs. 28%; 2-year RFS 93.5% vs. 92.4%; 5-year RFS 88.3% vs. 87.2%	Clinical utility	Tie et al. (2022); Tie et al. (2025)
Chaudhuri et al., lung cancer	Longitudinal cohort, n = 40	Post-treatment MRD	ctDNA detectable in 94% of evaluable recurrent cases; 72% before imaging; median lead time 5.2 months	Prognostic validity	Chaudhuri et al. (2017)
TRACERx, NSCLC	Longitudinal cohort, n = 197; 1,069 plasma samples	Postoperative surveillance	Landmark ctDNA+ in 25%; additional relapse detection during serial surveillance; median lead time 119 days	Prognostic/evolutionary monitoring	Abbosh et al. (2023)
Garcia-Murillas et al., breast cancer	Prospective cohort, n = 55	Molecular relapse	Postsurgical ctDNA: HR 25.1; median lead time 7.9 months	Prognostic validity	Garcia-Murillas et al. (2015)
c-TRAK TN	Prospective interventional surveillance, n = 161 under surveillance	TNBC molecular relapse	ctDNA detected in 44/161 (27.3%) within 12 months; feasibility demonstrated but intervention window limited	Feasibility; clinical utility unproven	Turner et al. (2023)
IMvigor010 ctDNA analysis	Biomarker analysis, n = 581 evaluable	Urothelial cancer	ctDNA + observation HR 6.3; ctDNA + atezolizumab vs. observation: DFS HR 0.58, OS HR 0.59	Prognostic + predictive	(Powles et al., 2021)
IMvigor011	Prospective randomized ctDNA-selected trial; 250 ctDNA + randomized	Adjuvant immunotherapy	DFS 9.9 vs. 4.8 months, HR 0.64; OS 32.8 vs. 21.1 mo, HR 0.59	Clinical utility	Powles et al. (2025)

## 2 Cellular origins, release, processing, and detection of plasma cfDNA

### 2.1 Cellular contributors and tissue context

Plasma cfDNA is a composite biological analyte rather than a tumor-specific product. Methylation-based tissue deconvolution has consistently shown that hematopoietic cells account for a large proportion of circulating DNA in both healthy individuals and patients with cancer (Mattox et al., 2023; Sun et al., 2015; Lehmann-Werman et al., 2016; Moss et al., 2018). Importantly, Mattox et al. analyzed cfDNA methylation profiles from 178 patients with pancreatic, colorectal, lung, or ovarian cancer and 64 individuals without cancer and found that leukocytes contributed approximately 76% of plasma cfDNA, with neutrophils representing a major source, even in samples with markedly elevated total cfDNA concentrations (Mattox et al., 2023). The excess cfDNA observed in many patients with cancer therefore did not originate predominantly from malignant cells or the corresponding normal epithelial tissue. These findings demonstrate that total cfDNA concentration cannot be interpreted as a direct surrogate for tumor burden and highlight the need to distinguish changes in tumor fraction from changes in background hematopoietic DNA.

The relative contribution of each compartment depends on more than cell number. Tissue architecture, vascular access, proliferative and death rates, inflammatory injury, immune-mediated killing, metastatic location, and prior treatment can all alter the probability that intracellular DNA reaches plasma. Highly vascular or advanced tumors generally shed more ctDNA than small localized tumors, whereas sanctuary sites and some tumor histologies may remain poorly represented in blood despite clinically relevant disease (Khier and Lohan, 2018; Bettegowda et al., 2014). These biological differences help explain why a negative plasma result may reflect limited shedding rather than absence of residual cancer.

### 2.2 Release mechanisms and fragment generation

cfDNA enters the circulation predominantly through cell death and subsequent nuclease-dependent processing, although necrosis, immune-mediated cytotoxicity, phagocytic processing, treatment-induced tissue injury, and potentially active release pathways may also contribute (Wan et al., 2017). These mechanisms generate molecularly distinct fragment populations rather than a uniform pool of extracellular DNA. Apoptotic chromatin cleavage produces the characteristic nucleosome-associated fragment distribution, whereas more disruptive forms of cell death can generate broader fragment-size profiles. Experimental studies further demonstrate that nucleases participate directly in determining plasma-DNA architecture: loss of DNASE1L3 alters both fragment-length distributions and end-motif frequencies, while DNASE1L3 and caspase-activated DNase contribute to the generation of circulating DNA fragments (Serpas et al., 2019; Watanabe et al., 2019).

Fragment size also carries information about tumor origin. Tumor-derived fragments are, on average, enriched among shorter cfDNA molecules, and fragment-size selection can improve recovery of mutant tumor DNA from plasma. Genome-wide fragmentation, nucleosomal positioning, preferred end coordinates, and end-motif patterns therefore provide biological information that complements mutation-based detection rather than merely representing technical properties of sequencing libraries (Khier and Lohan, 2018; Snyder et al., 2016; Cristiano et al., 2019; Jiang et al., 2020; Mouliere et al., 2018).

### 2.3 Circulating processing and systemic clearance

The plasma concentration of cfDNA represents the net balance between release and clearance. Once DNA fragments enter the circulation, they undergo further nuclease degradation and removal through phagocytic uptake and hepatic, splenic, and renal pathways (Khier and Lohan, 2018). Experimental and clinical kinetic studies indicate that circulating DNA can turn over within minutes to hours, although the apparent half-life varies according to fragment characteristics, disease state, tissue injury, and sampling design. This rapid turnover is one reason why ctDNA can respond earlier than radiographic measurements to changes in tumor biology, but it also creates sensitivity to perioperative injury, inflammation, organ dysfunction, and treatment-related cell death.

Accordingly, neither an isolated rise in ctDNA nor an early decline should automatically be equated with proportional tumor growth or complete eradication of viable malignant cells. Release and clearance can change simultaneously after surgery, radiotherapy, systemic therapy, infection, or tissue injury. Longitudinal measurements obtained at biologically appropriate time points therefore provide substantially more interpretable information than isolated plasma values.

### 2.4 Chromatin biology underlying methylation and fragmentomics

Methylation and fragmentomic assays are not merely technical alternatives to mutation detection; they read biological features established within the cell of origin. Tissue-specific DNA methylation is maintained through lineage programs and can identify the tissues contributing cfDNA even when recurrent tumor mutations are unavailable or extremely rare (Sun et al., 2015; Lehmann-Werman et al., 2016; Moss et al., 2018). Cancer-associated methylation changes may add another layer of discrimination, but their interpretation depends on the reference methylome, cell composition, and disease context.

Fragmentomic signals arise because cfDNA cleavage is constrained by chromatin accessibility, nucleosome positioning, transcriptional activity, and nuclease preference. Plasma DNA therefore retains nonrandom size distributions, nucleosome footprints, preferred genomic termini, and end motifs. Nucleosome footprints can inform tissue of origin, while genome-wide fragmentation and end-motif patterns can distinguish cancer-associated from nonmalignant cfDNA states (Snyder et al., 2016; Cristiano et al., 2019; Jiang et al., 2020). These signals are especially useful at low tumor fractions because information can be aggregated across many fragments rather than relying on a small number of mutant molecules. The biological origins, processing, and source-attribution framework of plasma cfDNA are summarized in Figure 2.

**FIGURE 2:**
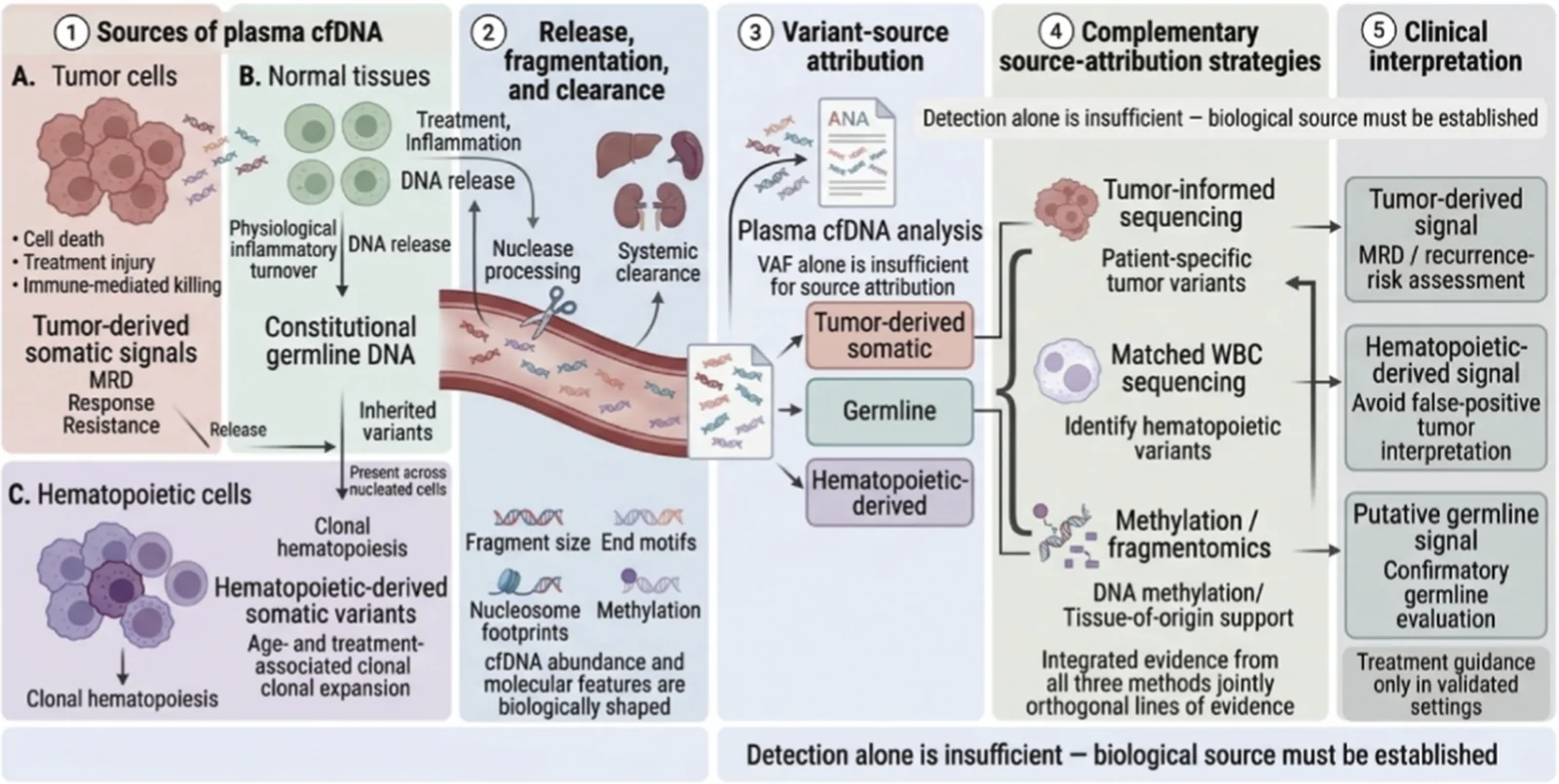
Biological origins, processing, and source attribution of plasma cfDNA. Plasma cfDNA represents a heterogeneous mixture derived from tumor cells, normal tissues, and hematopoietic cells. DNA release, nuclease processing, and systemic clearance shape cfDNA abundance and molecular features, including fragment size, end motifs, nucleosome footprints, and methylation patterns. Because variant allele fraction alone is insufficient to determine biological origin, accurate source attribution requires integration of complementary evidence, including tumor-informed sequencing, matched white blood cell sequencing, and methylation or fragmentomic features. Distinguishing tumor-derived, hematopoietic-derived, and putative germline signals is essential for appropriate interpretation of MRD and recurrence risk, avoidance of false-positive tumor calls, and referral for confirmatory germline evaluation when indicated. Created in BioRender. Zhang, B. (2026) https://BioRender.com/35mvpsx.

## 3 Variant-source attribution in plasma cfDNA: tumor-derived, germline, and hematopoietic signals

### 3.1 Tumor-derived somatic variation

Detection of a somatic variant in plasma does not by itself establish that the variant originated from a solid tumor. The same specimen can contain tumor-derived DNA, constitutional germline alleles, hematopoietic somatic variants, and low-frequency technical errors. Variant allele fraction alone is therefore an unreliable source discriminator, particularly in low-tumor-fraction settings in which true ctDNA and clonal-hematopoietic variants may occur at overlapping allele frequencies (Reinert et al., 2019; Razavi et al., 2019; Merker et al., 2018).

Tumor-informed assays reduce this ambiguity by restricting plasma analysis to variants demonstrated in the resected or biopsied tumor, especially when matched normal sequencing is incorporated during assay development. Plasma-only approaches require additional evidence, including genomic context, longitudinal kinetics, fragment length, methylation-based tissue-of-origin signatures, and matched leukocyte DNA. Source attribution should therefore be regarded as a probabilistic integration of orthogonal information rather than a binary inference based on allele fraction alone (Sun et al., 2015; Moss et al., 2018; Snyder et al., 2016; Cristiano et al., 2019; Razavi et al., 2019).

### 3.2 Germline findings: source attribution and confirmatory testing

Because most plasma cfDNA originates from nonmalignant cells, particularly hematopoietic cells, plasma sequencing inevitably samples a substantial component of the patient’s constitutional genomic background. Consequently, a pathogenic or likely pathogenic variant detected at a variant allele fraction approaching 50% may raise suspicion of a heterozygous germline alteration, but VAF alone is not sufficient to establish germline origin. The observed VAF is affected by the circulating tumor fraction, tumor purity, copy-number alterations, loss of heterozygosity (LOH), somatic amplification, subclonality, and clonal hematopoiesis. A near-50% VAF should therefore be regarded as a screening clue rather than a diagnostic threshold.

The ESMO Precision Medicine Working Group has developed a germline-focused framework for pathogenic variants identified during tumor genomic profiling and recommends dedicated germline follow-up rather than assigning constitutional status from tumor sequencing alone (Mandelker et al., 2019). The updated ESMO analysis of 49,264 paired tumor-normal samples further identified BRCA1, BRCA2, PALB2, MLH1, MSH2, MSH6, and RET as particularly actionable cancer-susceptibility genes for which germline follow-up should be considered irrespective of tumor type, while also extending consideration to genes such as ATM and CHEK2 (Kuzbari et al., 2023). Although these recommendations were primarily developed for tumor-detected variants, their underlying principle is directly relevant to plasma-only profiling, in which constitutional DNA represents a major component of the sequenced material.

The ESMO Precision Medicine Working Group recommendations for ctDNA assays similarly support cautious source attribution because plasma genotyping can contain tumor-derived, germline, and hematopoietic signals simultaneously. A pathogenic variant identified in ctDNA should therefore not be presumed to be germline solely because its VAF approaches 50% (Pascual et al., 2022). When tissue is available, comparison with the tumor profile can provide important contextual information, including assessment of LOH, copy-number changes, and concordant tumor alterations; however, tumor detection alone does not constitute formal proof of germline origin.

Accordingly, a suspected pathogenic or likely pathogenic germline variant detected by liquid biopsy should be reported as a potential germline finding and should trigger an appropriate clinical-genetics pathway rather than being communicated as a confirmed inherited diagnosis. Formal validation should preferentially be performed using a dedicated normal DNA specimen, such as peripheral-blood leukocyte DNA or saliva according to local genetics practice, with informed consent and genetic counseling. If clonal hematopoiesis or constitutional mosaicism is a plausible alternative explanation, interpretation may require additional genetics expertise and, in selected circumstances, testing of an alternative non-tumor specimen. Confirmation of germline status is particularly important because the result may affect targeted therapy, cancer surveillance, risk-reducing interventions, and cascade testing of relatives.

### 3.3 Clonal hematopoiesis as a parallel evolutionary system

Clonal hematopoiesis represents an independent somatic evolutionary process in hematopoietic stem and progenitor cells and is a major biological confounder of plasma genotyping. Its prevalence rises sharply with age. In a sequencing analysis of 12,380 individuals, Genovese et al. detected clonal hematopoiesis in approximately 10% of individuals older than 65 years but in only about 1% of those younger than 50 years. A separate analysis of 17,182 individuals by Jaiswal et al. similarly demonstrated age-dependent increases in detectable hematopoietic clones, with frequencies of 9.5% at 70–79 years, 11.7% at 80–89 years, and 18.4% among individuals aged 90 years or older (Genovese et al., 2014; Jaiswal et al., 2014).

Cancer therapy further modifies this evolutionary background. In 8,810 patients with nonhematologic malignancies, Coombs et al. identified clonal hematopoiesis in approximately 25%, including presumptive leukemia-driver mutations in 4.5%; clonal hematopoiesis was associated with increasing age, prior radiation exposure, and smoking, whereas PPM1D and TP53 mutations were enriched after chemotherapy (Coombs et al., 2017). Thus, hematopoietic clones should not be treated as a fixed sequencing artifact but as dynamic biological populations that can evolve under cytotoxic and inflammatory selection pressures.

The practical implication for ctDNA-MRD is substantial. Paired plasma and white-blood-cell sequencing has demonstrated that a substantial proportion of variants detected in plasma can originate from hematopoietic clones rather than the solid tumor, particularly when the tumor-derived fraction is low. High-intensity sequencing of matched plasma, tumor tissue, and leukocyte DNA has therefore established clonal hematopoiesis as a major source of apparent plasma somatic variation (Razavi et al., 2019). For high-specificity clinical interpretation, parallel sequencing of leukocyte DNA obtained from the buffy coat or peripheral blood should be regarded as the preferred reference strategy for CHIP subtraction whenever feasible. BloodPAC analytical-validation recommendations specifically identify sequencing of matched white blood cells at the same depth as plasma as a recommended approach for excluding non-tumor-associated CHIP variants, while emphasizing that purely bioinformatic CHIP filters require independent validation of sensitivity and specificity (Godsey et al., 2020).

Importantly, CHIP filtering should not rely on a simple gene-level blacklist because several genes recurrently altered in hematopoietic clones are also *bona fide* drivers of solid tumors. A practical approach is to flag a core set of CHIP-prone genes for matched-leukocyte review. These include the canonical age-related CHIP genes DNMT3A, TET2, and ASXL1; therapy- or DNA-damage-associated genes such as TP53 and PPM1D; and additional myeloid or spliceosome-associated genes including JAK2, SF3B1, SRSF2, U2AF1, ZRSR2, CBL, RUNX1, IDH1, IDH2, and BCOR. In patients previously exposed to cytotoxic therapy or radiotherapy, TP53 and PPM1D variants deserve particular scrutiny because these clones can be positively selected by treatment (Coombs et al., 2017). Plasma JAK2 variants, a proportion of TP53 variants, and occasional KRAS variants have been directly demonstrated to originate from clonal hematopoiesis rather than the solid tumor (Hu et al., 2018).

Detection of the same alteration in tumor tissue does not invariably establish tumor origin. Leukocyte admixture within tumor specimens can introduce hematopoietic variants into tumor-only sequencing data, creating a “CHIP-in-tissue” interpretative pitfall. In a large matched tumor-blood analysis, clonal-hematopoietic mutations were detectable in tumor specimens and could have been incorrectly classified as tumor-derived in the absence of matched blood sequencing (Ptashkin et al., 2018). Consequently, variants in genes shared between solid tumors and clonal hematopoiesis—particularly TP53, KRAS, NRAS, and IDH1/2—should not be automatically discarded or automatically assigned to the tumor. Their origin should instead be resolved by integrating matched leukocyte sequencing, tumor genotype and purity when available, variant allele fraction, treatment history, and longitudinal plasma kinetics.

### 3.4 Assay design, analytical sensitivity, and low-tumor-fraction detection

Assay design determines not only analytical sensitivity but also the degree to which biological source attribution is possible. Digital PCR and droplet digital PCR provide high sensitivity for a limited number of predefined alterations, whereas error-corrected next-generation sequencing can integrate multiple tumor-specific variants, phased variants, broad genomic panels, or genome-wide signals. Molecular barcoding, background-error modeling, and multi-locus integration have progressively improved detection of very low tumor fractions (Newman et al., 2014; Newman et al., 2016; Kurtz et al., 2021; Zviran et al., 2020).

Tumor-informed approaches first identify somatic alterations in tumor tissue, ideally using matched normal DNA, and subsequently track a patient-specific set of variants in plasma. This strategy increases specificity and reduces confusion with germline or hematopoietic variants but requires tissue access, initial sequencing, individualized assay design, and additional turnaround time (Reinert et al., 2019; Razavi et al., 2019; Chen et al., 2021). By contrast, tumor-uninformed approaches use fixed genomic panels, methylation signatures, fragmentomics, or multiomic classifiers and can be deployed without prior tumor sequencing. Their scalability is offset by a greater requirement for rigorous biological source attribution because nonmalignant tissues and hematopoietic clones can contribute overlapping molecular signals (Moss et al., 2018; Cristiano et al., 2019; Razavi et al., 2019; Parikh et al., 2021).

Analytical sensitivity must also be distinguished from clinical sensitivity. For low-level ctDNA assays, analytical validation should define the limit of blank, limit of detection, and, where quantitative reporting is intended, limit of quantification. These parameters are influenced by plasma volume, cfDNA input, the number of tracked loci, error-correction strategy, and positivity algorithm. A technically low detection threshold therefore does not guarantee high clinical sensitivity, because residual disease may remain undetectable as a consequence of limited tumor shedding, anatomical site, treatment, or sampling time (Pascual et al., 2022; Merker et al., 2018; Lockwood et al., 2023; Stetson et al., 2024).

## 4 ctDNA-MRD after curative-intent treatment: evidence validity by tumor type

After curative-intent treatment, ctDNA-MRD testing seeks evidence of occult malignant cells when conventional imaging shows no measurable disease. A positive result generally has strong prognostic validity, whereas a negative result means only that residual disease was not detected by a particular assay at a particular time. Interpretation should therefore separate three questions: whether ctDNA predicts recurrence, whether it predicts benefit from a specific treatment, and whether changing treatment according to ctDNA improves patient outcomes (Pascual et al., 2022; Verschoor et al., 2024; Kobayashi et al., 2025).

### 4.1 Colorectal cancer

Colorectal cancer currently provides the most mature evidence base for postoperative ctDNA-MRD. In a prospective study of 125 patients with stage I–III colorectal cancer, Reinert et al. reported that ctDNA positivity approximately 30 days after surgery was associated with a sevenfold higher risk of recurrence (HR 7.2, 95% CI 2.7–19.0). During longitudinal surveillance, ctDNA-positive patients had an even greater recurrence risk (HR 43.5, 95% CI 9.8–193.5), and molecular recurrence preceded standard radiographic detection by a mean of 8.7 months (Reinert et al., 2019; Tie et al., 2016; Tie et al., 2019). These data established that serial ctDNA is substantially more informative than a single postoperative measurement for recurrence-risk stratification.

The larger CIRCULATE-Japan GALAXY program strengthened this evidence. In the updated analysis of 2,240 patients with resectable colorectal cancer, ctDNA positivity within the postoperative MRD window was associated with markedly inferior disease-free survival (HR 11.99) and overall survival (HR 9.68). Among patients treated with adjuvant chemotherapy, sustained ctDNA clearance was associated with a 24-month DFS of 89.0%, compared with only 3.3% among patients with transient clearance (Kotani et al., 2023; Nakamura et al., 2024). These results demonstrate both the strong prognostic value of postoperative ctDNA and the additional information contained in longitudinal ctDNA kinetics.

Randomized evidence is available from DYNAMIC. Among 455 patients with stage II colon cancer, ctDNA-guided management reduced adjuvant chemotherapy use from 28% to 15% while maintaining noninferior 2-year recurrence-free survival (93.5% versus 92.4%). At approximately 5 years of follow-up, recurrence-free survival remained similar between ctDNA-guided and standard management (88.3% versus 87.2%), as did overall survival (93.8% versus 93.3%) (Tie et al., 2022; Tie et al., 2025). Thus, DYNAMIC provides direct clinical-utility evidence for treatment de-escalation in this defined population rather than simply demonstrating prognostic association.

### 4.2 Non-small cell lung cancer

Post-treatment ctDNA also has strong prognostic validity in localized NSCLC, although interventional evidence remains less mature. Using CAPP-seq in 40 patients with localized lung cancer, Chaudhuri et al. detected ctDNA in the first post-treatment sample in 94% of evaluable patients who subsequently experienced recurrence. Molecular detection preceded radiographic progression in 72% of these patients, with a median lead time of 5.2 months. TRACERx subsequently evaluated 1,069 longitudinal plasma samples from 197 patients with resected NSCLC. ctDNA was detected within 120 days after surgery in 25% of patients, including 49% of those who ultimately relapsed; serial surveillance at approximately 3–6-month intervals identified impending relapse in an additional 20% of patients who were initially landmark-negative. The overall median molecular lead time was 119 days (Chaudhuri et al., 2017; Abbosh et al., 2023). These data illustrate the benefit of serial testing but also demonstrate incomplete sensitivity, particularly in biologically indolent or low-shedding tumors. Therefore, postoperative ctDNA in NSCLC currently provides robust risk information and a platform for trial enrichment, but it should not yet be equated with universally validated treatment guidance.

### 4.3 Breast cancer

Early breast cancer illustrates both the potential and the limitations of ctDNA surveillance. In a prospective cohort of 55 patients, Garcia-Murillas et al. showed that post-treatment ctDNA detection was strongly associated with metastatic relapse, with a hazard ratio of 25.1 for a single postsurgical assessment and a median molecular lead time of 7.9 months using serial mutation tracking (Garcia-Murillas et al., 2015; Panet et al., 2024). However, prospective intervention remains challenging. In c-TRAK TN, 161 patients with moderate- or high-risk early triple-negative breast cancer entered prospective ctDNA surveillance, and 44 patients (27.3%) developed detectable ctDNA within 12 months. The trial demonstrated the feasibility of real-time molecular surveillance but also showed that ctDNA-triggered intervention may occur too late for some patients because radiographically detectable metastatic disease can already be present when molecular positivity is confirmed (Turner et al., 2023). Consequently, ctDNA in early breast cancer is currently better established as a marker of molecular relapse risk than as a validated trigger for routine systemic treatment.

### 4.4 Urothelial carcinoma

Urothelial carcinoma provides an instructive progression from retrospective biomarker association to prospective ctDNA-selected intervention. In the ctDNA analysis of IMvigor010, 214 of 581 evaluable patients (37%) were ctDNA-positive after surgery. Among patients managed by observation, ctDNA positivity was associated with markedly worse prognosis (HR 6.3). Within the ctDNA-positive subgroup, atezolizumab was associated with improved disease-free survival (HR 0.58, 95% CI 0.43–0.79) and overall survival (HR 0.59, 95% CI 0.41–0.86), whereas no comparable benefit was observed in ctDNA-negative patients (Bellmunt et al., 2021; Powles et al., 2021). Because these analyses were derived from biomarker-defined subgroups of a trial that was negative in the overall population, they provided compelling predictive evidence but did not alone establish clinical utility.

That distinction was subsequently addressed prospectively in IMvigor011. Among 761 enrolled patients undergoing serial postoperative ctDNA surveillance, 250 ctDNA-positive patients were randomized to atezolizumab or placebo. Median disease-free survival improved from 4.8 to 9.9 months (HR 0.64, 95% CI 0.47–0.87), while median overall survival improved from 21.1 to 32.8 months (HR 0.59, 95% CI 0.39–0.90). Among 357 patients who remained persistently ctDNA-negative, 2-year disease-free survival was 88% without empiric adjuvant immunotherapy (Powles et al., 2025). This trial provides direct evidence that ctDNA can be used prospectively to select a postoperative population for effective systemic intervention in a defined urothelial-cancer setting. The current evidence maturity of ctDNA-guided applications across representative solid tumors is summarized in Figure 3.

**FIGURE 3:**
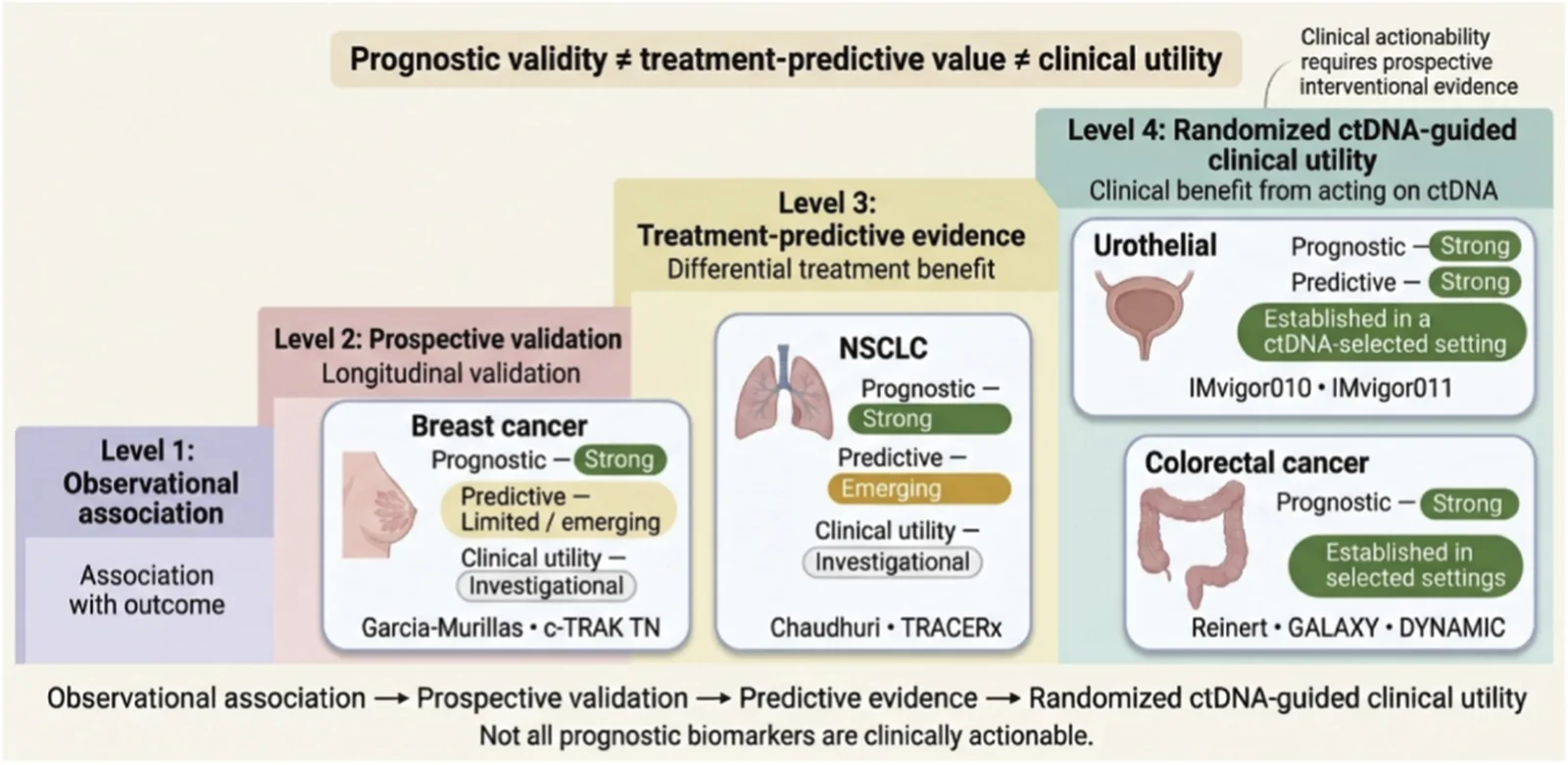
Evidence maturity of ctDNA-guided applications across solid tumors. This schematic illustrates the current evidence landscape for ctDNA-guided applications across representative solid tumors. The stepped framework reflects increasing levels of evidence, from observational association and prospective validation to treatment-predictive evidence and randomized ctDNA-guided clinical utility. The figure emphasizes that prognostic validity does not automatically translate into treatment-predictive value or clinical utility. Colorectal cancer and urothelial cancer currently show the strongest evidence for ctDNA-guided management in selected settings, whereas in NSCLC and breast cancer, ctDNA remains primarily a prognostic biomarker and its broader clinical utility is still investigational. Created in BioRender. Zhang, B. (2026) https://BioRender.com/f6db0pi.

### 4.5 Less common solid tumors and compartment-specific liquid biopsy

Although the most mature clinical evidence for ctDNA has been generated in common solid tumors, liquid biopsy may be particularly valuable in less common malignancies for which repeated tissue acquisition is difficult, risky, or limited by insufficient biopsy material. Biliary tract cancers (BTCs), including intrahepatic and extrahepatic cholangiocarcinoma, represent an important example because genomic profiling can identify therapeutically relevant alterations such as FGFR2 fusions or rearrangements, IDH1 mutations, ERBB2 alterations, BRAF alterations, and microsatellite instability, while adequate tissue may not always be available. In a cohort of 102 patients with advanced BTC, ctDNA profiling showed 84.8% sensitivity and a 79.4% positive predictive value relative to tissue genomic profiling and identified potentially targetable alterations in 34.3% of patients (Hwang et al., 2025). A separate molecular analysis of BTC demonstrated partially distinct but complementary genomic landscapes between circulating and tissue tumor DNA, supporting the use of ctDNA as an adjunct when tissue is limited and as a potential means of capturing spatial or temporal tumor heterogeneity (Astier et al., 2024).

The optimal liquid-biopsy compartment also depends on tumor anatomy. For primary central nervous system tumors and CNS metastases, plasma ctDNA may be limited by low systemic shedding and anatomical barriers, whereas cerebrospinal fluid (CSF) can provide greater access to tumor-derived DNA. CSF cell-free tumor DNA has been investigated for molecular diagnosis, identification of actionable genomic alterations, monitoring of therapeutic response, and assessment of intracranial tumor heterogeneity (Seoane et al., 2019). These observations emphasize that “liquid biopsy” should not be considered synonymous with plasma alone and that the biological compartment closest to the tumor may offer superior molecular information in selected malignancies.

This concept extends to other disease-specific biofluids and clinical settings. Aqueous-humor cfDNA tumor fraction, for example, has been associated with therapeutic response in retinoblastoma, illustrating the potential of locally derived fluid biomarkers in tumors for which conventional tissue sampling is problematic (Polski et al., 2020). Experimental head-and-neck cancer models have further demonstrated that longitudinal cfDNA kinetics can track tumor burden and recurrence (Muhanna et al., 2017). In metastatic melanoma, serial plasma ctDNA combined with functional imaging has provided complementary information on disease burden and genomic heterogeneity during treatment (Wong et al., 2017). Collectively, these studies illustrate that the clinical utility of liquid biopsy depends not only on assay sensitivity but also on selecting the most informative biological fluid and matching the biomarker strategy to the anatomical and evolutionary characteristics of each tumor type.

## 5 ctDNA as an interventional and longitudinal biomarker

The studies summarized above define an evidence continuum rather than a uniform standard of care. Postoperative ctDNA positivity is a reproducible prognostic marker across multiple solid tumors, but only a subset of settings has progressed from risk stratification to prospective treatment guidance. This distinction is central to interpretation because identification of a high-risk population does not demonstrate that intensifying therapy will improve outcome, while a negative ctDNA result does not automatically establish that effective standard treatment can be omitted.

At present, the clearest randomized evidence for ctDNA-directed management includes de-escalation of adjuvant chemotherapy in stage II colon cancer and selection of ctDNA-positive patients for adjuvant immunotherapy after surgery for muscle-invasive bladder cancer (Tie et al., 2022; Tie et al., 2025; Powles et al., 2025). By contrast, postoperative escalation, molecular-relapse treatment, and routine ctDNA-triggered treatment switching in NSCLC and breast cancer remain predominantly investigational. Future interventional studies should therefore prespecify whether ctDNA is intended for escalation, de-escalation, molecular-relapse therapy, response-adaptive switching, or clinical-trial enrichment and should evaluate disease-free survival, overall survival, treatment exposure, toxicity, and quality of life rather than molecular clearance alone.

### 5.1 Sampling time and serial interpretation

Sampling time is a biological component of the assay rather than a purely logistical choice. Immediately after surgery, tissue injury and inflammation can increase non-tumor cfDNA and dilute low-level ctDNA, whereas delayed sampling may miss an early opportunity for risk-adapted treatment. Representative studies have therefore used defined postoperative windows: DYNAMIC based treatment assignment on samples obtained at 4 and/or 7 weeks after surgery, while GALAXY defined an MRD window of approximately 2–10 weeks before adjuvant therapy when given (Kotani et al., 2023; Nakamura et al., 2024; Tie et al., 2022; Tie et al., 2025). These windows should be reported as study-specific designs rather than universal standards.

Serial testing provides information that a single postoperative sample cannot. In resected NSCLC, longitudinal surveillance at approximately 3–6-month intervals detected molecular recurrence in some patients who were initially ctDNA-negative (Abbosh et al., 2023). In c-TRAK TN, surveillance began after completion of standard treatment and was performed at approximately 3-month intervals, illustrating a different schedule adapted to breast-cancer relapse biology (Turner et al., 2023). The value of serial testing lies in identifying persistent negativity, clearance, persistence, or reappearance rather than treating one measurement as definitive.

### 5.2 Molecular lead time and clinical benefit

Molecular recurrence can precede radiographic recurrence in colorectal cancer, NSCLC, and breast cancer (Reinert et al., 2019; Chaudhuri et al., 2017; Abbosh et al., 2023; Garcia-Murillas et al., 2015). However, analytical lead time is not equivalent to clinical benefit. Earlier detection is useful only if it enables an effective intervention or a meaningful change in management. Without a validated response strategy, repeated positive tests may lead to additional imaging, anxiety, invasive procedures, or treatment with uncertain benefit. Surveillance studies should therefore report not only the interval between ctDNA detection and imaging, but also whether acting during that interval improves survival, quality of life, or treatment burden.

### 5.3 Response monitoring

During systemic therapy, early ctDNA decline or clearance can function as a pharmacodynamic correlate of response. In metastatic gastrointestinal cancers, changes at approximately 4 weeks predicted later imaging response, and normalization at approximately 1 month in metastatic colorectal cancer was associated with longer progression-free and overall survival (Parikh et al., 2020; Ghidini et al., 2025). Similar associations have been reported during immunotherapy in advanced solid tumors, melanoma, and NSCLC (Bratman et al., 2020; Lee et al., 2017; Seremet et al., 2019; Anagnostou et al., 2023). These findings support prognostic or response validity, but a single early plasma value should not replace imaging, symptoms, pathology, or established treatment criteria until response-guided switching is prospectively validated.

Presurgical ctDNA clearance during neoadjuvant therapy may also reflect treatment activity. In resectable NSCLC, exploratory analyses linked molecular clearance with favorable outcomes, but pathological response, resection findings, and radiographic assessment remain the established measures for perioperative decision-making (Forde et al., 2022; Forde et al., 2025). Molecular clearance should therefore be described as a complementary correlate rather than a validated surrogate endpoint.

### 5.4 Treatment-driven tumor evolution

Treatment provides a mechanistic bridge between MRD, response monitoring, and resistance surveillance. Effective therapy reduces sensitive clones and may transiently increase DNA release through cell death, whereas persistent disease reflects incomplete eradication. Continued therapeutic pressure can then select resistant subclones, changing both the abundance of ctDNA and the spectrum of variants released into plasma. A longitudinal plasma profile is therefore an evolutionary readout: clearance may indicate contraction of detectable clones, persistence may indicate residual resistant disease, and newly emerging variants may identify clonal selection.

This model is supported by resistance monitoring across tumor types. In EGFR-mutant NSCLC, plasma can identify EGFR T790M and later resistance alterations such as EGFR C797S or MET amplification (Oxnard et al., 2016; Ramalingam et al., 2018). In estrogen receptor-positive breast cancer, ESR1 mutations can emerge during endocrine therapy before clinical progression (Chu et al., 2016; Fribbens et al., 2018). In metastatic colorectal cancer, RAS-mutant clones can expand during anti-EGFR therapy and decline after drug withdrawal, creating a biological rationale for plasma-selected rechallenge (Misale et al., 2012; Sunakawa et al., 2020; Zaanan et al., 2025). These examples show that ctDNA is not merely a binary marker of disease presence; it can track the changing clonal composition produced by treatment selection.

Accordingly, serial ctDNA should be interpreted together with treatment history, expected shedding, imaging, symptoms, and available interventions. The clinical question is not only whether ctDNA is detectable, but which clone produced the signal, how that clone has changed under therapy, and whether a validated action follows from the result.

## 6 Challenges for implementation and analytical harmonization

Clinical implementation begins with control of preanalytical and analytical variation. EDTA tubes are appropriate when plasma can be separated promptly, whereas cell-stabilizing tubes are preferable when processing is delayed because they reduce leukocyte lysis and the release of genomic DNA. Delayed plasma separation, incomplete cell removal, or suboptimal centrifugation can increase high-molecular-weight genomic DNA contamination and dilute low-level ctDNA signals. Plasma storage conditions should be standardized, and repeated freeze-thaw cycles should be minimized. cfDNA extraction method, plasma volume, DNA input, error suppression, and reporting thresholds can further alter the observed signal. Assays should therefore be validated for a defined intended use, with standardized specimen handling and transparent reporting of analytical performance (Merker et al., 2018; Stetson et al., 2024).

False-negative results remain a major concern in low-volume disease. A negative ctDNA result may reflect a genuine absence of detectable tumor-derived DNA, limited biological shedding, or technical factors such as insufficient cfDNA recovery or suboptimal specimen quality. Total cfDNA yield alone is not sufficient to establish specimen adequacy, because an apparently high yield may partly reflect leukocyte-derived genomic DNA contamination. Quality assessment should therefore consider fragment-size distribution, evidence of high-molecular-weight genomic DNA contamination, and the amount of amplifiable or otherwise informative DNA entering the assay. These factors should be considered together with tumor type, disease burden, metastatic site, treatment, and sampling time when interpreting a negative MRD result (Pascual et al., 2022; Kobayashi et al., 2025; Bettegowda et al., 2014). This distinction is especially important when considering de-escalation of effective therapy.

False-positive interpretation can arise from clonal hematopoiesis, constitutional germline variants, or technical artifacts. Matched white blood cell sequencing, tumor-informed assay design, orthogonal tissue-of-origin evidence, and confirmatory germline testing reduce but do not eliminate this risk (Moss et al., 2018; Razavi et al., 2019; Schrader et al., 2016; Mandelker et al., 2017; Raymond et al., 2016; Tung et al., 2024). Reports should clearly separate likely tumor-derived findings from suspected hematopoietic or germline findings and should acknowledge unresolved source attribution.

Quantitative ctDNA descriptors should also be kept distinct. Variant allele fraction (VAF) represents the proportion of analyzed molecules carrying a specific variant; absolute mutant copies describe the number or concentration of mutant molecules; tumor fraction estimates the overall proportion of cfDNA derived from tumor cells; and molecular positivity is a binary classification based on assay-specific thresholds or algorithms. These measures are influenced by assay design, plasma and DNA input, background cfDNA concentration, target selection, error correction, and bioinformatic rules. Numerical ctDNA values should therefore not be compared directly across platforms or studies without methodological harmonization.

Bioinformatic processing should be considered an integral component of assay validation rather than a hidden downstream step. A clinically interpretable ctDNA pipeline should transparently report read-level quality control, alignment and duplicate handling, unique molecular identifier (UMI) family construction and consensus generation when applicable, background-error suppression, minimum molecular coverage, variant-calling thresholds, germline and clonal-hematopoiesis filtering, and procedures for estimating tumor fraction and detecting copy-number alterations or structural variants. Software, reference genome, relevant databases, and pipeline versions should also be documented to permit reproducibility and cross-study comparison. Analytical and statistical validation should define the limit of blank, limit of detection, and limit of quantification for the intended application and should assess false-positive rates, precision, reproducibility, and robustness across runs, operators, reagent lots, and clinically relevant DNA inputs. The AMP/CAP consensus recommendations provide a practical framework for validating and reporting these analytical characteristics in cfDNA assays (Lockwood et al., 2023).

Several methodological strategies illustrate how bioinformatic processing can improve sensitivity without simply lowering variant-calling thresholds. Molecular barcoding combined with integrated digital error suppression reduces stereotypical sequencing errors and improves detection of low-frequency ctDNA variants (Newman et al., 2016). Phased-variant approaches increase the effective specificity of MRD detection by requiring multiple tumor-associated substitutions to occur on the same DNA molecule (Kurtz et al., 2021). Genome-wide integration of large numbers of patient-specific mutations represents another strategy for increasing sensitivity at extremely low tumor fractions (Zviran et al., 2020). Machine-learning or composite classifiers should additionally use prespecified feature engineering, separated training and validation datasets, appropriate cross-validation, and preferably independent external validation to minimize overfitting and spectrum bias. Similar tissue-based molecular profiling studies in solid tumors have demonstrated the potential of transcriptomic or proteomic biomarkers for prognostic stratification and characterization of tumor immune features, further underscoring the importance of rigorous validation before clinical translation (Zhang et al., 2024; Wu et al., 2021).

Bioinformatic interpretation is also expanding beyond single-nucleotide variants and small insertions or deletions. Contemporary approaches increasingly combine mutation burden with copy-number profiles, methylation, fragment length, nucleosomal organization, end motifs, and other epigenomic features to estimate circulating tumor fraction and tissue of origin. Recent work has highlighted the potential value of integrating genomic and epigenomic information into multimodal ctDNA frameworks, particularly when individual signal classes become weak at low tumor fractions (De Paolis et al., 2025). Nevertheless, multimodal models introduce additional requirements for normalization, feature calibration, training-set representativeness, and independent validation before clinical transfer. Tumor-fraction estimation is also important for interpreting negative plasma results: in a large paired liquid-biopsy/tissue analysis, the negative predictive value for driver alterations increased markedly when ctDNA tumor fraction was ≥1%, whereas clinically relevant drivers were frequently recovered by tissue testing after negative plasma results with tumor fraction <1% (Rolfo et al., 2024). Thus, a negative liquid-biopsy result should be interpreted together with an estimate of sample informativeness, and reflex tissue profiling remains particularly important when plasma tumor fraction is low.

For clinical reporting, an interpretable ctDNA report should specify the assay methodology, specimen type and input, analytical detection capability, criteria for molecular positivity or negativity, relevant quality-control findings, reported metric(s), and key analytical limitations. The LoD should be stated for assays used for low-level detection, together with LoB and LoQ when relevant to the intended use. In particular, a ctDNA-negative result should be reported as “no tumor-derived signal detected within the validated analytical capability of the assay in this specimen” rather than as evidence that residual disease is absent. Such qualification is especially important in MRD testing, where clinical decisions may depend on signals near the lower boundary of assay detection.

Timing and seriality also require disease-specific standardization. Postoperative sampling must balance tissue-injury-related background against the need for timely adjuvant decisions, while surveillance intervals should reflect relapse biology and available interventions. Thresholds for molecular response, clearance, persistence, and recurrence remain assay- and context-dependent, and a single universal cutoff is unlikely to be appropriate across solid tumors.

Cross-platform harmonization is equally important for clinical implementation. Standardized reference materials spanning clinically relevant low ctDNA levels, interlaboratory proficiency testing or external quality assessment, and harmonized analytical validation frameworks are needed to assess reproducibility and comparability across laboratories. For longitudinal monitoring, the same validated platform should be maintained whenever feasible. If a platform change is unavoidable, bridging or comparability studies should be considered because differences in target design, DNA input, error-suppression strategies, bioinformatic pipelines, and positivity thresholds may mimic or obscure true changes in ctDNA kinetics.

Finally, implementation depends on turnaround time, tissue availability, cost, reimbursement, and access to genetics and oncology expertise. Tumor-informed assays offer high specificity but require tissue and individualized design, whereas tumor-naive multiomic approaches may be more scalable but place greater demands on biological source attribution. Cost-effectiveness will depend on assay price, test performance, treatment effect, and whether ctDNA-guided care changes outcomes in the intended population (Kramer et al., 2024).

## 7 Evidence gaps and future priorities

Despite rapid advances in analytical sensitivity, several barriers continue to separate ctDNA detection from routine clinical decision-making. First, improved sensitivity must not come at the expense of source specificity. At very low tumor fractions, clonal hematopoiesis, germline variants, background sequencing errors, and nonmalignant tissue injury become increasingly important competing sources of signal. Future MRD platforms should therefore integrate matched leukocyte analysis, tumor-informed variant selection, methylation or fragmentomic tissue-of-origin information, and explicit reporting of uncertain source attribution rather than relying on variant allele fraction alone.

Second, negative ctDNA results remain biologically and analytically context dependent. The probability of detection varies with tumor type, disease site, vascular access, plasma volume, the number of informative loci, postoperative timing, and assay characteristics. A universal interpretation of ctDNA negativity is consequently inappropriate. Disease-specific negative predictive values and serial-testing strategies will be required before ctDNA-negative status can be used broadly to omit established therapies.

Third, molecular lead time should not be treated as an endpoint in itself. Detecting recurrence months before imaging is clinically valuable only when an effective intervention exists and when earlier treatment improves patient-centered outcomes. Future trials should therefore move beyond demonstrating earlier molecular detection and directly test whether ctDNA-triggered intervention improves survival, reduces treatment burden, preserves quality of life, or increases the probability of curative salvage.

Finally, cross-platform harmonization will be essential for clinical implementation. Differences in blood collection, plasma volume, cfDNA input, target number, sequencing depth, error suppression, positivity algorithms, and reporting metrics can produce discordant results even when assays are applied to the same biological setting. Prospective head-to-head comparisons, standardized reference materials, external quality-assessment programs, and clearly defined intended-use populations are needed before ctDNA results can be interpreted consistently across laboratories and clinical trials.

## 8 Conclusions

Plasma cfDNA is generated by interacting malignant, normal-tissue, and hematopoietic compartments, and its abundance and molecular features are continuously reshaped by cell death, chromatin organization, nuclease processing, systemic clearance, treatment, and clonal evolution. Clinical ctDNA interpretation must therefore combine sensitive detection with reliable source attribution and an understanding of the biological context in which a plasma signal is generated.

The clinical evidence should also be interpreted hierarchically. Postoperative ctDNA positivity has strong prognostic validity across several solid tumors, but prognostic association does not by itself establish treatment benefit. Randomized evidence now supports ctDNA-guided management in defined settings, including chemotherapy de-escalation in stage II colon cancer and ctDNA-selected adjuvant immunotherapy in muscle-invasive bladder cancer. In contrast, routine ctDNA-triggered escalation, molecular-relapse treatment, and response-adaptive switching in many other solid tumors remain investigational.

Future progress will depend less on detecting ever smaller quantities of DNA in isolation and more on demonstrating that biologically accurate, analytically reproducible, and disease-specific ctDNA interpretation leads to better patient outcomes. The transition from ctDNA as a highly informative biomarker to a broadly actionable clinical tool will therefore require prospective intervention trials, improved source attribution, standardized analytical frameworks, and explicit evidence that acting on a molecular signal provides benefit beyond conventional clinical assessment.
